# Unnatural amino acid compounds as potent multi-target inhibitors of aldose reductase, α-glucosidase, and α-amylase: integrated in vitro, SAR, and molecular dynamics insights

**DOI:** 10.1007/s00210-026-05249-1

**Published:** 2026-03-25

**Authors:** Serpil Gerni, Cansu Öztürk, Songül Bayrak, Yeliz Demir, Ufuk Atmaca, Dejan Milenković, Dušan Dimić, Ömer İrfan Küfrevioğlu

**Affiliations:** 1 Department of Chemistry, Faculty of Science, Ataturk University, Erzurum, Turkey; 2 Department of Pharmacy Services Nihat Delibalta Göle Vocational High School, Ardahan University, 75700 Ardahan, Turkey; 3 Department of Science, Institute for Information Technologies, University of Kragujevac, Jovana Cvijića Bb, 34000 Kragujevac, Serbia; 4 Faculty of Physical Chemistry, University of Belgrade, Studentski Trg 12-16, 11000 Belgrade, Serbia

**Keywords:** *N*-methoxysulfonyl β-ketoester, Aldose reductase, Molecular dynamics, α-Glucosidase

## Abstract

Diabetes mellitus is a multifactorial metabolic disorder in which sustained post-prandial hyperglycaemia and aberrant activation of the polyol pathway contribute to disease progression and long-term complications. Simultaneous modulation of digestive enzymes and aldose reductase (ALR2) therefore represents a rational multitarget therapeutic strategy. In this study, a series of previously reported aryl-substituted unnatural *N*-methoxysulfonyl β-ketoester derivatives were investigated for their inhibitory potential against ALR2, α-glucosidase, and α-amylase. Compound **1i** exhibited the strongest ALR2 inhibition with K_i_ :0.493 ± 0.155 µM and IC_50_: 1.638 ± 0.44 µM. For α-glucosidase, compound **1h** showed the highest potency (K_i_ :1.341 ± 0.181 µM), while compound **1j** demonstrated strong α-amylase inhibition (IC_50_: 1.361 ± 0.26 µM). The compounds were evaluated through in vitro enzyme inhibition assays supported by comprehensive structure–activity relationship (SAR) analysis. Several derivatives displayed pronounced inhibitory activity, achieving sub-micromolar K_i_ values against ALR2 and low-micromolar inhibition of α-glucosidase and α-amylase, in some cases surpassing reference inhibitors. SAR analysis revealed that ALR2 inhibition is strongly governed by planar aromatic expansion and conformational rigidity, whereas α-glucosidase and α-amylase preferentially accommodate compact, hydrophobically enriched substituents, highlighting distinct steric and electronic requirements across targets. A strong correlation was observed between experimental and docking-derived K_i_ values, supporting the consistency of the computational protocol. Subsequent 100 ns molecular dynamics simulations confirmed the stability of the most active ligand–enzyme complexes under physiological conditions. Overall, this integrated experimental and computational evaluation identifies unnatural *N*-methoxysulfonyl β-ketoester derivatives as promising multitarget antidiabetic lead scaffolds.

**Supplementary Information:**

The online version contains supplementary material available at 10.1007/s00210-026-05249-1.

## Introduction

Diabetes mellitus (DM) is a continuum of metabolic diseases characterized by the lack of insulin resistance, insulin production, or a combination of the two pathophysiological processes. Such disruptions lead to prolonged hyperglycaemia and changes in carbohydrate, lipid, and protein metabolism (Sever et al. 2025). Insulin-dependent diabetes (type 1 diabetes, T1D) represents about 5–10% of all diabetes; the disease develops when pancreatic β-cells are destroyed, leaving a person without insulin. T1D can either be idiopathic or autoimmune-mediated (Bruggeman and Schatz 2023). On the other hand, type 2 diabetes (T2D) or non-insulin-dependent diabetes mellitus is a condition that is defined by resistance of cells to insulin activity (Mahgoub et al. 2023). Most diabetic patients are diagnosed with T2D, which is approximately 90 to 95% of patients. As per the latest International Diabetes Federation (IDF) statistics, 537 million individuals aged from 20 to 79 years old were diabetic across the world. They were projected to increase to 643 million and 783 million in 2030 and 2045 respectively (Hossain et al. 2024; Siam et al. 2024). To this end, diabetes has become a serious public health concern due to its associated comorbidities, including acute metabolic complications (Efeoglu et al. 2025; Tokalı et al. 2025a). Hypoglycaemic coma and ketoacidosis are acute metabolic sequelae, symptoms of dangerously high blood glucose. The complications of diabetes may be divided into microvascular (retinopathy, neuropathy, cataracts, and nephropathy) and macrovascular (cerebrovascular and cardiovascular diseases resulting in stroke and myocardial infarction) ones (Tokalı et al. 2025b). Diabetic neuropathy, which is characterized as abnormal or reduced sensitivity, has a stocking-glove type of distribution, first of the feet then extends to fingers and hands (Jensen 2023). Diabetic retinopathy is a retinal disease, which is manifested by the swelling of the macula and the development of non-normal retinal vessels that end up causing severe visual loss or blindness (Tang et al. 2024). Such complications are caused by prolonged hyperglycaemia that causes harm to the blood vessels and peripheral nerves (Lu et al. 2023).

Aberrant activation of the polyol pathway plays a key role in the pathogenesis of diabetes-related complications. The main process involved in this pathway involves a two-step metabolic process where glucose is reduced to sorbitol before being oxidized to fructose (Türkeş et al 2022a,b). This is catalyzed by two important enzymes, aldose reductase (ALR2), which uses NADPH as a cofactor to catalyze the conversion of glucose to sorbitol, and sorbitol dehydrogenase (SDH), which, in combination with NAD^+^ as a cofactor, oxidizes sorbitol to fructose (Türkeş et al. 2022,b). In hyperglycaemic conditions, the ALR2 is provoked by high glucose levels, thus switching glucose metabolism to the active polyol pathway. ALR2 plays a dual role: firstly, it transforms the toxic aldehydes produced by the reaction of reactive oxygen species (ROS) into less toxic alcohols, and secondly, it transforms the surplus glucose into sorbitol, which is oxidized to fructose (Zognjani et al. 2025). Subsequently, ALR2 activity modulation and polyol pathway is a future potential treatment option to counter the effects of diabetic complications (Güleç et al. 2025).

The enzymes that help in the digestion of dietary starch are α-amylase and α-glucosidase, which bring about the hydrolysis of starch and sugar into simple molecules of glucose (Li et al. 2025). In this respect, the inhibition of these enzymes has become an important form of therapy to decrease the level of post-prandial blood sugar by inhibiting the digestion of carbohydrates (Bhujle et al. 2025). The primary goal of antidiabetic agents targeting α-glucosidase and α-amylase is to reduce post-prandial hyperglycaemia. This approach is currently achieved using FDA-approved agents such as acarbose, voglibose, and miglitol. Acarbose functions as a dual α-glucosidase/α-amylase inhibitor, while voglibose and miglitol selectively inhibit α-glucosidase (Khirallah et al. 2022; Reddy and Aruna 2025). Nevertheless, such agents are linked to gastrointestinal adverse effects such as diarrhea, abdominal pain, flatulence, and bloating (Du and Zhao 2025). In addition to the decreasing post-prandial glycaemia, a drop in the level of HbA1c can be attained (Landgraf et al. 2026). For these reasons, considerable research efforts have been directed toward the identification of novel α-amylase and α-glucosidase inhibitors, many of which have been reported in recent years (Khan et al. 2024; Mai et al. 2024).

Unnatural amino acids (UAAs) hold great significance in inorganic synthesis, biochemistry, and materials science. The UAAs are produced to be incorporated into peptides, natural products, and pharmaceutical chemistry (Sharma et al. 2024). This has resulted in a wide variety of synthetic approaches to UAAs such as cross-coupling, enolate-based, C-H activation, imine reactivity, deaminative reactions, and enzymatic syntheses (Hegedus 1995). As highly efficient drug-like molecules, UAAs as structural motifs such as paclitaxel (PTX, an anticancer agent) (Blaskovich 2016), O-[^11^C]methyl-l-tyrosine (Tyr, a tumor-imaging agent) (Iwata et al. 2003), and 4-acetylphenylalanine (a component of an antibody–drug conjugate) (Axup et al. 2012) are important biologically active molecules of interest. Thus, there is significant significance of the novel and productive synthetic methods of UAAs and sulfamates. In our previous work, Abul et al. (2024) reported the synthesis of the aryl-substituted N-methoxysulfonyl β-ketoester derivatives and evaluated their inhibitory activity against several metabolic enzymes associated with oxidative stress and diabetes-related biochemical pathways. Although that study established the general multitarget inhibitory potential of this scaffold, enzymes directly involved in the polyol pathway and post-prandial carbohydrate digestion particularly ALR2, α-glucosidase, and α-amylase were not systematically investigated. The β-ketoester moiety provides a conjugated carbonyl system capable of forming hydrogen-bond interactions with catalytic residues and cofactors within enzyme active sites. Such carbonyl-containing fragments are frequently observed in ALR2 inhibitors where they interact with residues located in the anionic binding pocket. The presence of the sulfamate (–SO_2_NH–) functionality introduces both hydrogen-bond donor and acceptor capabilities, which may facilitate anchoring of the ligand within polar regions of enzyme active sites. Taken together, this combination of polar interaction sites and hydrophobic aromatic elements suggested that N-methoxysulfonyl β-ketoester derivatives could potentially engage both the catalytic and hydrophobic regions of these enzymes. These structural considerations provided the initial rationale for evaluating this compound library as potential multitarget inhibitors of ALR2, α-glucosidase, and α-amylase.

In addition to the new biological assays, the current study provides a detailed structure–activity relationship (SAR) analysis supported by multivariate statistical modeling (PCA), contour SAR mapping, and molecular dynamics simulations (MD), which were not included in the previous report. This approach enables a deeper mechanistic understanding of the structural determinants governing enzyme inhibition and establishes the compounds as potential multitarget antidiabetic leads. The synthesized compounds were thoroughly evaluated biologically by in vitro enzyme inhibitory assays which were supplemented by thorough 2D interaction profiling and molecular docking to describe the binding orientations in the catalytic pockets. Altogether, this experimental and computational tight collaboration was supposed to discover structurally optimized dual or triple-target anti-diabetic lead molecules with better potency, selectivity, and pharmacophoric relevance.

## Materials and methods

### Chemistry

**Fig. 1:**
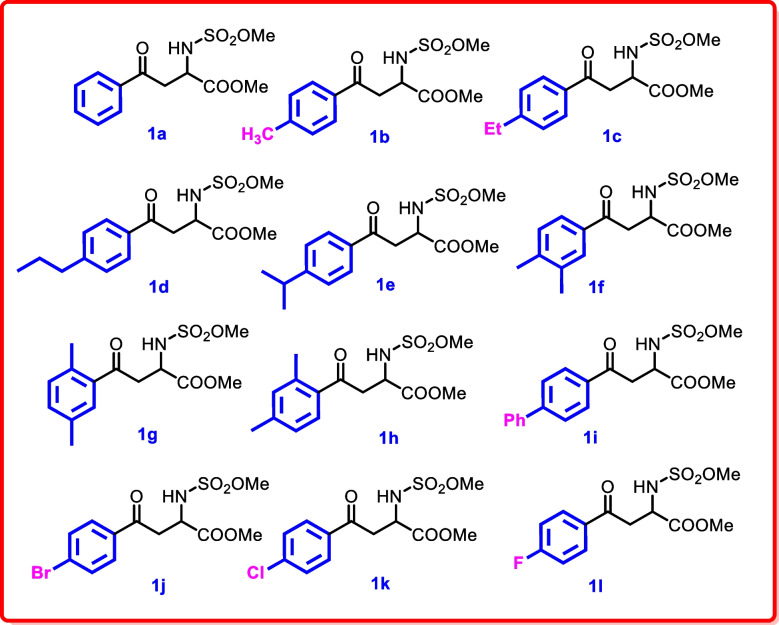
General chemical structures of the unnatural amino acid compounds used in this study

The synthesis and structural characterization of the unnatural amino acids (UAAs, **1a–l**) derivatives investigated in this study were previously reported by Abul et al. (2024). Therefore, the detailed synthetic procedures are not repeated here, and the compounds used in the present work were prepared according to the previously described protocol. This study presents all experimental details, analytical data, and spectral information for **1a–l** compounds. In this approach, an efficient, simple, and single-step method was used to obtain non-natural amino acids and their derivatives, starting from 4-oxo-4-phenylbut-2-enoic acid derivatives, via intramolecular aza-Michael addition in the presence of chlorosulfonyl isocyanate (CSI) and methanol (Fig. 1).

### Enzyme inhibition

#### ALR2, α-glucosidase, and α-amylase inhibition assay

Dimethyl sulfoxide (DMSO) was used to dissolve twelve early synthesized compounds and a standard compound, epelrastat (EPR) and acarbose (ACR), at an initial concentration of 1 mg/mL. The concentration of DMSO in the final reaction mixture was around 1%. A DMSO vehicle control experiment was performed in parallel for all enzymatic assays to verify that the solvent itself did not influence enzyme activity. At the final concentration used in the assay mixtures (approximately 1% v/v). Consistent with previous research, the kinetic examinations using (Cerelli et al. 1986) were used. Sheep kidney ALR2 was isolated from fresh sheep kidney tissue according to the procedure described by previous studies (Tokalı et al. 2023); Akdağ et al. 2025). The purified enzyme preparation was used in the inhibition assays at a final activity of 0.1 U/mL. The inhibitory impact of synthesized compounds (1a-l) on α-glucosidase (*Saccharomyces cerevisiae* was purchased from Sigma-Aldrich, catalog number: G5003 and used at a final concentration of 1 U/mL) was investigated using *p*-nitrophenyl-d-glycopyranoside (p-NPG) as the substrate, following the methodology reported (Tao et al. 2013). The absorbance values were quantified using spectrophotometry at a wavelength of 405 nm. The α-amylase activity was designated conforming to the procedure recorded by Xiao et al. (2006). The inhibitory mechanisms of these compounds against ALR2, α-glucosidase, and α-amylase *in vitro* were examined*.* To determine IC_50_ values, a series of inhibitor concentrations were tested, and a dose–response curve was plotted. Each measurement was performed in triplicate, and data were expressed as mean ± SD. Lineweaver–Burk curves were created for all compounds to identify K_i_ constants and their inhibition types. The polydisperse character of starch prevents accurate determination of [S] in molar units, thereby limiting the validity of double-reciprocal kinetic plots for reliable K_i_ derivation. For this reason, IC_50_ values were considered more appropriate for comparative evaluation of α-amylase inhibition within the compound series.

### Principal component analysis (PCA)-based multivariate analysis

PCA was performed in R statistical software (R Foundation for Statistical Computing, Vienna, Austria) to explore the multivariate relationships between physicochemical descriptors and enzyme inhibitory activities. Electronic (Hammett σ), steric (Taft *E*s), and lipophilicity (logP) descriptors were compiled for each compound together with the experimentally determined inhibition constants (K_i_) against ALR2 and α-glucosidase. Prior to analysis, all continuous variables were standardized using z-score normalization** (**mean = 0, standard deviation = 1) to eliminate scale effects. PCA was conducted using the correlation matrix with eigen decomposition. The number of principal components retained for interpretation was determined based on eigenvalues (> 1) and scree plot inspection. The first two principal components (Dim-1 and Dim-2), accounting for the majority of total variance, were selected for visualization and interpretation. Score plots and loading vectors were jointly examined to assess the relative contribution of steric, electronic, and lipophilic parameters to enzyme inhibition profiles.

### Contour SAR mapping for ALR2 and α-glucosidase

To visualize the combined influence of steric and electronic factors on enzyme inhibition, two-dimensional contour structure–activity relationship (SAR) maps were constructed. Hammett σ constants and Taft Es values were used as independent variables, while experimental K_i_ values for ALR2 and α-glucosidase served as dependent response variables. Interpolated contour surfaces were generated using grid-based smoothing to depict regions associated with favorable and unfavorable inhibitory activity. Low-K_i_ regions were interpreted as zones of enhanced binding affinity, whereas high- K_i_ regions indicated reduced activity. Separate contour maps were generated for ALR2 and α-glucosidase to capture enzyme-specific steric and electronic preferences and to facilitate direct comparison of their distinct binding requirements.

### Substituent effects and selectivity analysis

To quantify enzyme selectivity, selectivity indices were calculated using the ratios K_i_(α-glucosidase)/K_i_(ALR2) for ALR2 selectivity and K_i_(ALR2)/K_i_(α-glucosidase) for α-glucosidase selectivity. Compounds were grouped according to substituent class (alkyl, aryl, halogenated, bulky, and unsubstituted), and the distribution of selectivity indices within each class was evaluated. Box-plot analysis was employed to visualize intra-class variability and median selectivity trends. This approach enabled systematic assessment of how steric bulk and electronic characteristics of substituents modulate enzyme preference and multitarget behavior.

### Molecular docking

#### Ligand preparation

Ligand structures (**1a–1l**) were optimized using density functional theory (DFT) in Gaussian 16 (Frisch et al. 2016) with the M062X functional (Walker et al. 2013) and 6–311 + + G(d,p) basis set, ensuring stable minima (no imaginary frequencies). Optimized structures were converted to PDBQT format using OpenBabel (O'Boyle et al. 2011) for docking.

#### Receptor preparation

Crystal structures of the receptors (PDB IDs: 3WY1, 1US0, and 1B2Y) were retrieved from the RCSB Protein Data Bank in PDB format (Nahoum et al. 2000; Howard et al. 2004; Shen et al. 2015). Receptors were prepared by removing co-crystallized ligands, water molecules, and cofactors using Discovery Studio 4.0 (BIOVIA Discovery Studio 2021. Dassault Systèmes, San Diego, CA). AutoDockTools (Morris et al. 2009) was used to add polar hydrogens and calculate Kollman partial charges. Protonation states of titratable residues were adjusted to approximate physiological pH (7.4). The prepared receptors were saved in PDBQT format.

#### Docking setup

Docking grids were defined using AutoGridFR (Ravindranath et al. 2015; Zhang et al. 2019) with grid centers (in Å): 3WY1 (α-glucosidase): − 0.161, − 18.336, 17.472; 1US0 (ALR2): 22.220, − 8.819, 22.581; 1B2Y (α-amylase): 20.521, 4.551, 48.186.

For α-glucosidase, the docking receptor was the GH31 enzyme from *Halomonas* sp. H11 (PDB ID: 3WY1). This structure was chosen as a high-resolution GH31 surrogate with a clearly defined catalytic machinery and overall fold that are conserved across GH31 α-glucosidases, including yeast and mammalian enzymes. Although 3WY1 is of bacterial origin and its active-site residues are not identical to those of the *Saccharomyces cerevisiae* or human intestinal maltase-glucoamylase (MGAM), it provides a structurally well-characterized template that enables a mechanistic, qualitative analysis of ligand binding in a GH31-type active site. Docking was performed with AutoDock-GPU (Santos-Martins et al. 2021) in rigid mode (200 genetic algorithm runs, –nrun 200; –nev 2,500,000; –ngen 42,000; ADADELTA local search; population size 150). Binding energies are reported in kcal/mol. Poses were visualized in PyMOL (Molecular Graphics System, Version 3.0. Schrödinger, LLC). Binding energies (Δ*G*) were converted to K_i_ (μM) using Δ*G* = RT ln K_i_ at 298 K. Calculated K_i_ values are reported as approximate IC_50_ equivalents for qualitative comparison with experimental data.

### Molecular dynamics

Docked complexes **1h**–α-glucosidase, **1i**–ALR2, and **1j**–α-amylase were used as starting models for 100 ns MD simulations. Protein was described with CHARMM36 force field (Best et al. 2012), while the small molecule ligands (**1a–1l**, EPR, ACR) were parameterized using the CHARMM General Force Field (CGenFF), generated via CHARMM GUI (Lee et al. 2016), solvated with TIP3P water, and neutralized with 0.15 M KCl. Energy minimization was followed by NVT equilibration (2 ns) and NPT production run using GROMACS 5.1.5 (Abraham et al. 2015) with LINCS constraints, modified Berendsen thermostat (τ_T_ = 1 ps), and Parrinello–Rahman barostat (τ_P_ = 2 ps) (Hess et al. 1997).

## Results and discussion

### Chemistry

The tested compounds were prepared according to the previously reported metal-free one-pot protocol based on chlorosulfonyl isocyanate (CSI)–mediated intramolecular aza-Michael cyclization of 4-oxo-4-arylbut-2-enoic acid derivatives (Abul et al. 2024). Briefly, the corresponding enone acids were converted to the target sulfamate-containing unnatural amino acid derivatives under mild conditions using catalytic triflic acid in dichloromethane at room temperature, followed by methanol addition; the transformation is proposed to proceed via activation of the carboxyl group, intramolecular aza-Michael addition, and final decarboxylation to afford the methyl 2-((methoxysulfonyl)amino)−4-oxo-4-arylbutanoates in generally good to excellent yields. The structures of the products were confirmed by standard spectroscopic methods (e.g., ^1^H/^13^C NMR and HRMS) (Abul et al. 2024). Accordingly, in the present work, these literature compounds were employed as the chemical platform for our enzyme inhibition assays.

### ALR2 inhibition

For a clearer SAR interpretation, the ALR2 inhibition data were analyzed according to substituent classes rather than individual compounds. In this framework, the present series can be broadly grouped into four categories: alkyl-substituted derivatives, aryl-expanded derivatives, halogenated derivatives, and disubstituted derivatives (Fig. 1). The SAR trends discussed below are therefore interpreted primarily on the basis of experimental K_i_ values within each substituent class. The progressive increase in the degree of inhibition within the series suggests that even minor alterations in the size of aryl substituents, the hydrophobic surface area, the planarity, and the electronic nature have a strong effect on the affinity of the said molecules to the enzyme. By using fold-difference calculations to compare the quantitative contribution of each structural feature relative to the reference inhibitor of EPR (K_i_ :0.97 µM, Table 1) as the baseline, the quantitative contribution of each structural feature can easily be identified. The inhibition type of the reference inhibitor EPR was evaluated using Lineweaver–Burk plots, which were consistent with a non-competitive inhibition mechanism under the assay conditions used.

**Table 1:** IC_50_ and K_i_ values of unnatural amino acid compounds on ALR2, α-glucosidase, and α-amylase enzyme activity

IC_50_(μM)									K_i_(μM)	
Compounds	ALR2	R^2^		α-Glu	R^2^	α-amylase	R^2^		ALR2	α-Glu
**1a**	3.537 ± 0.99	0.998		7.700 ± 0.98	0.976	2.863 ± 0.39	0.985		1.475 ± 0.402	3.08 ± 0.720
**1b**	2.179 ± 0.87	0.981		2.851 ± 0.54	0.983	3.628 ± 0.68	0.972		1.224 ± 0.265	3.439 ± 1.328
**1c**	5.680 ± 0.34	0.988		3.223 ± 0.37	0.989	6.026 ± 1.54	0.978		1.957 ± 0.490	1.469 ± 0.637
**1d**	5.290 ± 0.55	0.982		5.923 ± 0.78	0.988	2.615 ± 0.23	0.975		10.625 ± 4.541	2.275 ± 0.744
**1e**	6.794 ± 0.34	0.981		2.431 ± 0.39	0.985	13.075 ± 1.79	0.977		6.498 ± 2.723	2.043 ± 0.614
**1f**	1.698 ± 0.31	0.983		11.177 ± 1.45	0.969	4.682 ± 0.89	0.970		4.216 ± 0.718	3.467 ± 1.713
**1g**	2.341 ± 0.77	0.966		4.985 ± 0.99	0.971	2.457 ± 0.56	0.990		10.553 ± 1.543	3.471 ± 1.346
**1h**	6.537 ± 0.13	0.978		2.272 ± 0.46	0.984	1.513 ± 0.51	0.988		11.944 ± 5.783	1.341 ± 0.181
**1i**	1.638 ± 0.44	0.978		6.537 ± 0.32	0.993	3.253 ± 1.03	0.983		0.493 ± 0.155	4.243 ± 1.394
**1j**	3.107 ± 0.56	0.988		5.775 ± 1.03	0.959	1.361 ± 0.26	0.985		2.729 ± 1.242	6.258 ± 1.709
**1k**	2.707 ± 0.87	0.971		11.745 ± 1.68	0.984	3.666 ± 0.78	0.986		2.086 ± 0.936	2.051 ± 1.001
**1l**	3.647 ± 0.60	0.962		2.186 ± 0.34	0.988	5.727 ± 0.54	0.973		1.610 ± 0.507	3.256 ± 0.539
**EPR**^a^	0.866 ± 0.14	0.962							0.97 ± 0.087	
**ACR**^b^	-		-	2.80		10.00				12.60 ± 0.78

^a^Epelrastat (EPR) was used as a positive control for the ALR2 enzyme

^b^Acarbose (ACR) was used as a positive control for α-glycosidase and α-amylase enzymes, which was taken from reference (Taslimi et al. 2018)

Compound **1i** stands out as the strongest ALR2 inhibitor with a K_i_ value of 0.493 µM, which is one that is 1.97 times stronger than the standard (Table 1). This sharp increase is mostly due to the biphenyl substituent which increases significantly the aromatic surface area without altering the overall planarity of the molecule. It is assumed that the extended π-system can expand the hydrophobic contact interface of the molecule, allowing more desirable desolvation and closer complementarity with the hydrophobic environment of ALR2. These findings are very strong indications that the enzyme is more attracted to the substituents that can expand the planar coverage of aromatics instead of the ones that add non-planar or sterically challenging bulk. Therefore, the unusual inhibitory potential of **1i** is likely to be through the interactive effect of aromatic extension, rigidity of conformation, and higher surface density. It should also be noted that the interpretation of SAR trends was performed with consideration of the experimental variability associated with K_i_ determinations. Therefore, when the inhibitory constants of certain derivatives fall within partially overlapping uncertainty ranges, the corresponding compounds were considered to exhibit broadly comparable activity rather than strictly different potency levels. Consequently, the SAR discussion focuses primarily on larger and more consistent activity shifts within the series rather than on small numerical differences that may lie within the experimental error margins.

As shown in Fig. 1, compound **1a** contains an unsubstituted phenyl ring and can therefore serve as a structural baseline for evaluating substituent effects. Introduction of small substituents on the aromatic ring, as observed in compounds **1b** and **1c**, leads to moderate changes in inhibitory activity, indicating that the enzyme binding pocket can tolerate limited steric modification. In contrast, compound **1d** bearing an alkyl-type substituent shows a noticeable decrease in inhibitory potency, suggesting that non-aromatic substituents reduce the ability of the ligand to establish stabilizing π–π or hydrophobic interactions within the active site. These comparisons highlight the importance of maintaining an aromatic substituent for optimal ligand–enzyme interactions.

In the case of the halogen substituted derivatives, the effect of hydrophobic volume and substituent polarizability are seen as a combination of intermediate inhibitory behaviour. Compound **1l** (K_i_ :1.610 µM), the chloro analog **1k** (K_i_ :2.086 μM) and the bromo analog **1j** (K_i_ 2.729 µM) are found to be less inhibitory, and it is possible that excessive electronegativity does not promote hydrophobic stabilization and can even reduce affinity by changing the local electronic distribution (Table 1). However, despite being weaker than the reference inhibitor EPR (K_i_ :0.97 μM), these halogenated derivatives still display comparatively favorable inhibition within the series, indicating that halogen substitution when accompanied by appropriate steric balance can confer a measurable advantage relative to many non-halogenated analogs. The influence of halogen substituents on ALR2 inhibition can also be interpreted in the context of the enzyme’s hydrophobic selectivity pocket. This region, formed by residues such as Trp111, Tyr48, Phe122, and Trp219, is known to accommodate aromatic moieties through hydrophobic and π–π interactions. Introduction of halogen atoms can enhance binding affinity through several mechanisms. Halogen substitution increases molecular polarizability and hydrophobic surface area, which may strengthen van der Waals contacts within the selectivity pocket. In contrast, highly electronegative substituents such as fluorine may alter the electronic distribution of the aromatic ring without substantially increasing steric bulk, which can modulate π-stacking interactions within the pocket (Kousaxidis et al. 2023, 2025a,b).

Recent medicinal chemistry studies on ALR2 inhibitors have similarly reported that halogenated aromatic scaffolds often improve affinity toward the ALR2 specificity pocket by enhancing hydrophobic complementarity and stabilizing aromatic interactions within the catalytic site. Therefore, the moderate activity observed for the halogenated derivatives in the present series is consistent with previously reported SARs for halogen-containing ALR2 inhibitors.

There is a sharp loss in potency in compounds with aliphatic substituents, which highlights the importance of aromaticity in the inhibition of ALR2. Compounds **1d** (K_i_ :10.625 µM) and **1g** (K_i_ :10.553 µM) are more than ten times weaker than the reference inhibitor and more than 21 times weaker than **1i** (Table 1). These findings suggest that non-aromatic substituents significantly affect the efficiency of binding. Aliphatic groups are also π-surface area, highly flexible in their conformational features, and do not align with the hydrophobic scaffolds preferred by the enzyme; they do not thus place the β-ketoester scaffold in an energetically accessible conformation and thus incur substantial affinity losses.

Intermediate potencies are noted on derivatives with small hydrophobic substituents on the aromatic ring. Compound **1f** (K_i_ :4.216 µM) is slightly less active than the reference (equivalent to about 4.35-fold), indicating that though the methyl substitution also moderately raises the local hydrophobic density, this effect is too small to counterbalance the fairly limited aromatic surface (Table 1). Compound **1h** (K_i_ :11.94 µM) shows markedly reduced inhibitory activity compared with other derivatives in the series. This effect cannot be attributed to disruption of aromatic planarity, since methyl substitution does not alter the inherent planarity of the aromatic ring. Instead, the 2,4-dimethyl substitution pattern likely introduces steric interactions with residues located within the ALR2 specificity pocket, which may force the ligand to adopt a suboptimal binding orientation and thereby weaken stabilizing π–π and hydrophobic interactions with aromatic residues in the active site.

Taken together, these results indicate a systematic logic of structuring: the maximum of ALR2 inhibition is provided by substituents that increase planar aromatic contact area, increase the density of hydrophobic interactions, and preserve conformational rigidity, and non-aromatic, excessively electronegative, or sterically disruptive groups cause significant reduction of inhibitory activity. Docking analysis further supports this interpretation, showing that the aromatic substituents of the most potent derivatives occupy the hydrophobic specificity pocket while the polar functional groups remain oriented toward the catalytic residues responsible for substrate recognition.

### α-Glucosidase inhibition

As with ALR2, the α-glucosidase inhibition data were interpreted using a substituent-class framework. The compounds were grouped as alkyl-substituted, aryl-expanded, halogenated, and disubstituted derivatives (Fig. 1), allowing a clearer comparison of how different substituent types influence inhibitory potency within the same scaffold. The quantitative framework provided by the use of ACR (K_i_ :12.60 µM) as the standard reference gives the possibility to compare the structural contributions of aryl substituents to the binding affinity (Table 1). The distribution of the K_i_ values among the series indicates that the α-glucosidase inhibition relies heavily on the steric compactness, the hydrophobic density, the symmetry of the substitutions and degree of spatial compactness of the aryl groupings, with the enzyme showing a unanimous preference for moderately sized, hydrophobic aryl groups that are spatially compact. Compound **1h** exhibits the greatest inhibitory ability of all derivatives with a K_i_ of 1.341 µM, which is an impressive 9.40-fold stronger than ACR (Table 1). Such a spectacular performance can be defined by the fact that the 2,4-dimethylphenyl substituent produces a structurally compact but hydrophobically enriched aryl environment. The two methyl groups raise the local surface density but leave the substituents symmetrically bound, and the molecule is then free to adopt a low-strain position that increases binding complementarity. The high affinity of **1h** shows that α-glucosidase prefers substituents that enhance hydrophobic cohesion without significantly expanding the steric footprint. The **1h** methyl groups inhibit rotational motions and enhance structural rigidity and minimize the entropic penalty during binding and stabilization of the interaction between the inhibitor and the enzyme. The **1e** (K_i_ 2.043 µM) and **1c** (K_i_ :1.469 µM) inhibitors, with a 6.17-fold and 8.58-fold increase over the standard, respectively, are the most potent inhibitors after **1h** (Table 1). Those aryl substituents of theirs, ethyl in **1e** and a small electronic adjustment in **1c**, provide a desired steadiness of hydrophobic expansion without overstraining the sterics. These findings underscore the fact that α-glucosidase is responsive to moderate levels of hydrophobic augmentation in which substituents marginally raise surface reactivity and retain the entire molecular size. It is because the single substituent pattern of **1c** exhibits a pronounced potency that electronic modulation of a planar aromatic system can be effective in increasing the alignment of the ligand, which, in turn, raises binding affinity by making small changes to hydrophobic distribution. Intermediate inhibitory activities were reported with compounds **1l** (K_i_ :3.256 µM), **1a** (K_i_ :3.08 µM), **1b** (K_i_ 3.439 µM), and **1f** (K_i_ :3.467 µM) with 3.63 to 4.09-fold greater potency compared to ACR (Table 1). The structural properties of these molecules are compatible with desirable glucose binding properties, which are a moderate size in the aroma, limited conformational flexibility and aryl-plane substituents that do not extensively expand the molecule or cause significant distortions. The comparable activities of **1a** and **1l** suggest that α-glucosidase can accommodate small to moderately sized hydrophobic substituents at the para position, such as fluorine, without impairing binding, whereas bulkier groups that disrupt the continuity of the aromatic surface or introduce steric congestion are less well tolerated. The synergistic activity of **1a, 1b**, and **1f** supports the idea that structural simplicity with compact hydrophobicity can be used to obtain significant α-glucosidase inhibition. Oppositely on the spectrum, there are a few derivatives with significantly lower activity that prove the strict structural requirements of the enzyme. Compounds **1i** (K_i_ :4.243 µM) and **1j** (K_i_ :6.258 µM) are much less potent with only 2.97 and 2.01-fold stronger than ACR (Table 1). The two compounds are characterized by the excess of aromatic expansion, biphenyl in **1i** and bromophenyl in **1j,** making the steric envelope larger than the size that α-glucosidase can accept. Such observations suggest that carbohydrate hydrolysed enzymes prefer compact aromatic substituents and disfavor excessively extended systems of π-systems, which bring in steric incompatibility. In the case of **1i**, the biphenyl moiety will be large and the conformational penalties that inhibit efficient ligand accommodation and reduce inhibitory potency are likely to occur. Therefore, the aromatic expansion is helpful in the ALR2 case but not in the case of α-glucosidase. Although compounds **1d** (K_i_ :2.275 µM) and **1k** (K_i_ :2.051 µM) exhibit inhibitory activity at the lower end of the potency range within this compound series, both derivatives remain substantially more potent than the clinical reference inhibitor ACR (K_i_ 12.60 µM). This observation indicates that even the less active members of the series maintain significant inhibitory potency against α-glucosidase relative to the standard drug. However, when comparing derivatives with closely related K_i_ values, the uncertainty associated with the experimental measurements was taken into account. In cases where the confidence intervals of the inhibition constants overlapped, the compounds were interpreted as belonging to a similar activity tier rather than representing statistically meaningful potency differences. This approach ensures that the SAR interpretation emphasizes robust structural trends instead of minor numerical variations. Overall, the experimental α-glucosidase data indicate that compact hydrophobic substituents are generally more favorable than extensive aromatic expansion. In particular, several disubstituted derivatives showed strong inhibition, whereas excessive aromatic enlargement did not consistently improve activity. Thus, for α-glucosidase inhibition in this scaffold, balanced hydrophobic substitution appears to be more advantageous than maximizing aromatic surface area.

### α-Amylase inhibition

Α-amylase inhibition data of the synthesized unnatural amino acid compounds indicate a very coherent, structurally explanable SAR pattern when the activities are considered in strict terms of their IC_50_ values. ACR (IC_50_: 10.00 µM), as a reference inhibitor, gives a definite quantitative parameter against which changes in the size of aryl substituent, the hydrophobic mass, the conformational rigidity, and the steric efficiency can be evaluated with regard to inhibitory potency (Table 1). The findings show a selective preference of α-amylase for compact but hydrophobically enriched aromatic substituents, and the penalty of excessive steric extension and substituent patterns which disrupt structural correspondence of the ligand in the binding cavity.

The IC_50_ of **1a** was 2.863 µM of phenyl, and thus, the parent scaffold is inherently well-fitted in the α-amylase active site. This compound can thus be considered as being a structurally and pharmacologically viable template, where the aromatic ring presents a suitable hydrophobic surface and the carbonyl and methoxysulfonylamino groups are well oriented to form hydrogen-bond and polar interactions with catalytic residues. Introduction of a para-methyl substituent, as in compound **1b**, leads to a modest decrease in activity, reflected by an IC_50_ of 3.628 µM (Table 1).

The extra methyl group makes the region more hydrophobic; however, it seems to add steric bias, which slightly disturbs the preferred orientation of the phenyl ring in the binding cleft. Therefore, non-specific para-methylation does not have a significant reinforcing effect on binding and can cause a small conformational cost to a ligand-enzyme complex. In the case of the para position of the compound occupied by an ethyl group in compound **1c**, the inhibitory potency becomes even lower, with an IC_50_ being 6.026 µM. The increased steric encumbrance provided by the longer alkyl chain does not provide commensurate benefits in favorable dispersion contacts, thus decreasing complementarity to the active site. Interestingly, the para-propyl derivative **1d** exhibits a recovery and even a slight increase in activity compared to the parent company, and the IC_50_ is 2.615 µM (Table 1). This non-monotonic curve in the para-alkyl series indicates that conformational freedom and the accurate spatial projection of the substituent are at least as significant as the mere size of the steric. It is reasonable to think that the propyl group in **1d** can relax to decrease steric clash and still bind the hydrophobic subpocket better than the ethyl group in **1c**. Conversely, the para-isopropyl analog **1e** exhibits a strong loss of activity, having an IC_50_ of 13.075 µM and is therefore the least active in the series and weaker than ACR (IC_50_ : 10 µM) (Table 1). The branched isopropyl unit is likely to exert a large steric burden to the para position and disrupt the orientation of the aromatic core and to favor unfavorable binding.

The dimethyl-substituted analogs **1f, 1g,** and **1h** resonate better with steric and electronic effects. The intermediate activity pattern exhibited by compound **1f** that has a 3,4-dimethyl substitution pattern with an IC_50_ of 4.682 µM. Though it possesses a lower potency than **1a**, it remains stronger than the reference inhibitor ACR, which suggests that further methyl groups can be retained to retain useful potency. More significant improvements are seen in the case of compounds **1g** and **1h**, which have 2,5- and 2,4-dimethyl substitution patterns and have IC_50_ values of 2.457 and 1.513 µM, respectively (Table 1). The presence of an ortho methyl group in the molecules is also likely to cause a controlled torsion of the aromatic ring, preorganizing the scaffold into a conformation where the carbonyl and sulfonamide groups are better positioned to access key residues in the catalytic pocket. At the same time, higher electron density on the ring and larger hydrophobic surface area can enhance π–pi and CH–pi hydrogen bonding with aromatic amino acids. Of the dimethyl analogs, **1h** comes out as one of the strongest in the series.

The biphenyl analog **1i**, which possesses an IC_50_ of 3.253 µM and is strong, is still more effective than ACR. Extensive hydrophobic and π stacking interactions should be favored by the extended π-system; this should be offset by the further rotational freedom of the biphenyl unit entailing costly entropic requirements at least in the context of the potency levels achieved by the most effective dimethyl and halogen-substituted analogs. Of special interest is the action of the halogenated derivatives **1j**, **1k**, and **1l**. In the series, para-bromo compound **1j** has the lowest IC_50_ value (1.361 µM), suggesting that a large, polarizable bromine atom provides a highly favorable contact topography, presumably a combination of high hydrophobic contacts with the nucleophilic residues and potential halogen-bonding. The para-chloro derivative **1k**, IC_50_ of 3.666 µM, and the para-fluoro analog **1l**, IC_50_ of 5.727 µM, are relatively active, though not as active as **1j** and **1h** (Table 1). This trend in this series indicates that the larger the size of the halogen and the higher the polarizability, the better the binding, and the bromine offers the best balance of steric fit and electronic contribution.

### SAR analysis

#### PCA-based multivariate interpretation

To avoid overinterpretation of marginal activity differences, SAR trends were interpreted together with the uncertainty/confidence-interval plot derived from the experimental inhibition data. In this framework, potency differences were considered mechanistically meaningful only when they were clearly larger than the associated experimental variability. Accordingly, compounds with partially overlapping uncertainty intervals were treated as having comparable activity, and the corresponding structural effects were discussed as tentative rather than definitive. This more conservative interpretation provides a more statistically grounded basis for distinguishing robust SAR patterns from small numerical differences within the experimental error range.

**Fig. 2:**
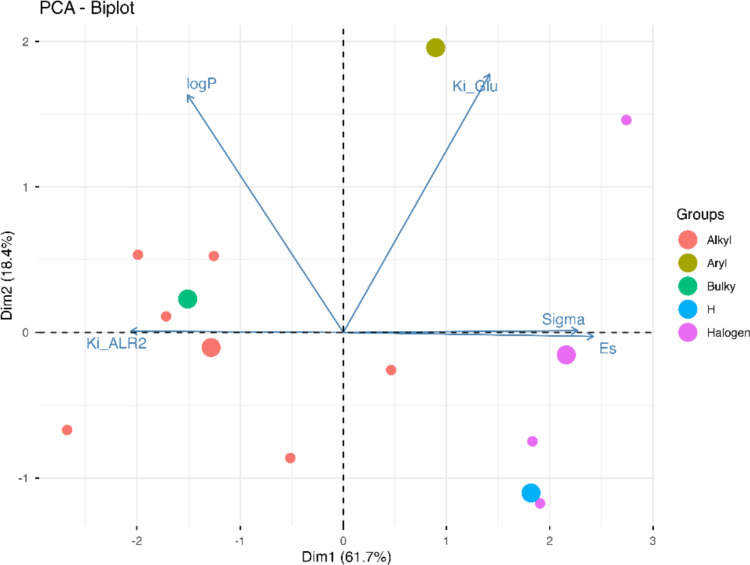
PCA biplot integrating physicochemical descriptors and inhibitory effects against ALR2 and α-glucosidase

To combine the electronic (Hammett σ), steric (Taft Es) and lipophilicity (log P) descriptors with the experimentally measured inhibitory constants (K_i_) of ALR2 and α-glucosidase, a PCA was conducted. The initial two major constituents contributed a significant percentage of the total variance thus indicating that the chemical and biological variation of the synthesized compounds is sufficiently represented in a low-dimensional space. A loading of the vectors indicated that both Hammett-sigma and Taft-Es played a significant role in Dim-1 and indicated that electronic and steric effects are the most influential in determining enzymatic inhibition. By contrast, log P, which is the effect caused by lipophilicity, was the key affecting Dim2, indicating a secondary and measurable effect of lipophilicity on activity. The distribution of compounds on the PCA map showed that halogenated and bulky compounds were clustering around the α-glucosidase activity vector, and small alkyl substituents were around the ALR2 vector. These results show that substituent class has a significant effect on the difference in activity profile between the two enzymes (Fig. 2).

#### Contour SAR mapping for ALR2 and α-glucosidase

To visualize how steric (Es) and electronic (σ) parameters affected ALR2 inhibition, two-dimensional contour SAR maps were created. The contour SAR map for ALR2 should be interpreted together with the experimental inhibition data presented in Table 1. In contrast to a simple steric-size interpretation, the experimental results indicate that ALR2 inhibition in this series is primarily driven by the presence of extended planar aromatic systems rather than by minimal steric bulk. The most potent inhibitor, compound **1i** (K_i_ 0.493 µM), contains a biphenyl substituent that significantly enlarges the aromatic surface area while maintaining planarity. This structural feature enables extensive π–π stacking and hydrophobic interactions within the tryptophan-rich specificity pocket of ALR2, which includes residues such as TRP79, TRP111, TRP219, and TYR48. Therefore, the improved potency observed for 1i can be attributed to enhanced aromatic stacking and hydrophobic complementarity rather than to reduced steric size. In contrast, derivatives bearing aliphatic substituents, such as 1 d and 1 g, show markedly reduced activity despite their relatively small steric footprint. This decrease in inhibition likely arises from the loss of planar aromatic interactions that are required to stabilize binding within the hydrophobic specificity pocket of ALR2. Consequently, the SAR analysis indicates that the key determinant of ALR2 inhibition in this compound series is the availability of a sufficiently extended and planar aromatic system capable of engaging in stabilizing π–π and hydrophobic contacts within the enzyme active site.

**Fig. 3:**
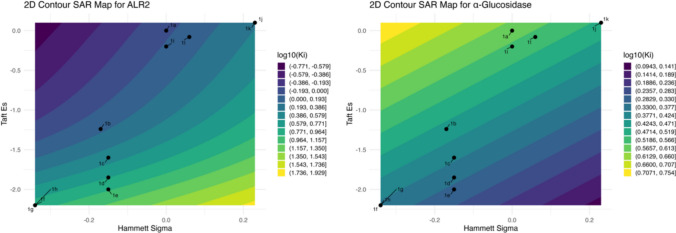
Two-dimensional contour SAR maps illustrating steric and electronic effects on ALR2 and α-glucosidase inhibition

An opposing steric profile was observed with the α-glucosidase. The relevant contour SAR map showed that the higher the steric bulk (the less negative Es values), the better was the inhibitory potency. This observation is in line with the broader and more hydrophobic binding pocket of the enzyme binding the bulky substituents that are bound in the additional hydrophobic regions and form stronger van der Waals interactions. The compounds with more massive substituents, **1a, 1i,** and **1l** were elevated in the bottom of K_i_ regions of the contour surface. The influence of the electronic effects (Hammett σ) on the activity of the α-glucosidase was found to be weaker than that of the ALR2, which supports the assumptions that steric complementarity is the dominant force. The conflicting steric demands between ALR2 and alpha-glucosidase can be used to explain the different selectivity patterns in the series (Fig. 3).

#### Effects of substituents on ALR2 and α-glucosidase selectivity

The complementary index SI_ALR2 = K_i_(α-Glu)/K_i_(ALR2) was used to analyze the selectivity of ALR2. Compared to α-glucosidase, it was found that compounds with hydrogen or small alkyl substituents were the most selective by ALR2. This trend can be explained by the high steric constraints of the ALR2 active site which is not favorable to large groups. Once more halogen substituents were found to form a coherent cluster with intermediate selectivity in relation to ALR2, which indicates an equal relationship between steric suitability and favorable electronic factors. Bulky substituents, which were highly active intoxicated with α-glucosidase, generated low-ALR2 selectivity, thus supporting the conflicting steric demands of the two enzymes. The substituent-class analysis, on the whole, demonstrates steric complementarity as the key determinant of enzyme preference.

**Fig. 4:**
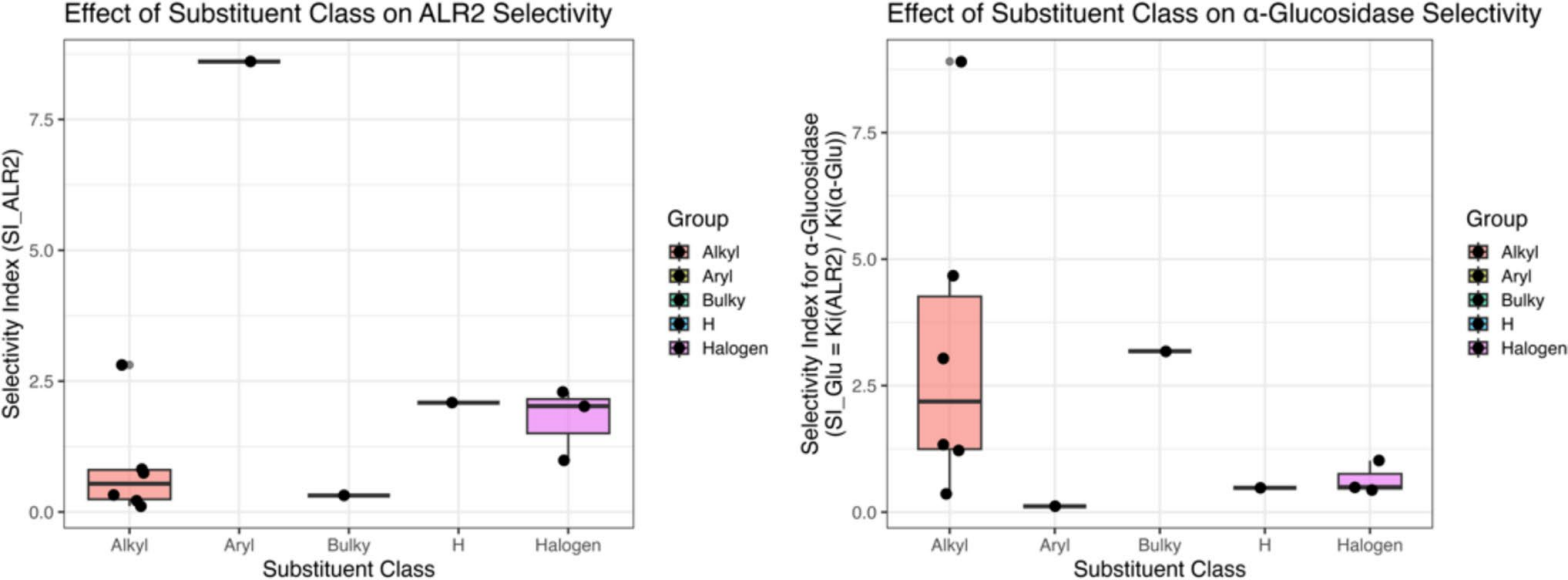
The effect of substituent class (alkyl, aryl, bulky, H, and halogen) on enzyme selectivity, presented as boxplots for ALR2 (left) and α-glucosidase (right)

Substituent class effect on α-glucosidase selectivity was assessed by means of the selectivity index SI-α-Glu = K_i_(ALR2)/K_i_ (α-Glu). Box-plot analysis established that the best selectivity of α-glucosidase was favored by bulky substituents which indicated their sterically complementary interactions with the larger catalytic site in the enzyme. Halogenated derivatives were also reasonably selective but more consistent, which is probably because of other halogen–π or dipole interactions that stabilized the binding of the ligands. By comparison, alkyl substituents yielded considerably different selectivity values indicating that any small steric effects within this group have a significant effect on α-glucosidase affinity. These findings support the argument that bulk of substituents is a crucial predictor of selectivity to α-glucosidase (Fig. 4).

#### Correlation analysis of multitarget enzyme inhibition

**Fig. 5:**
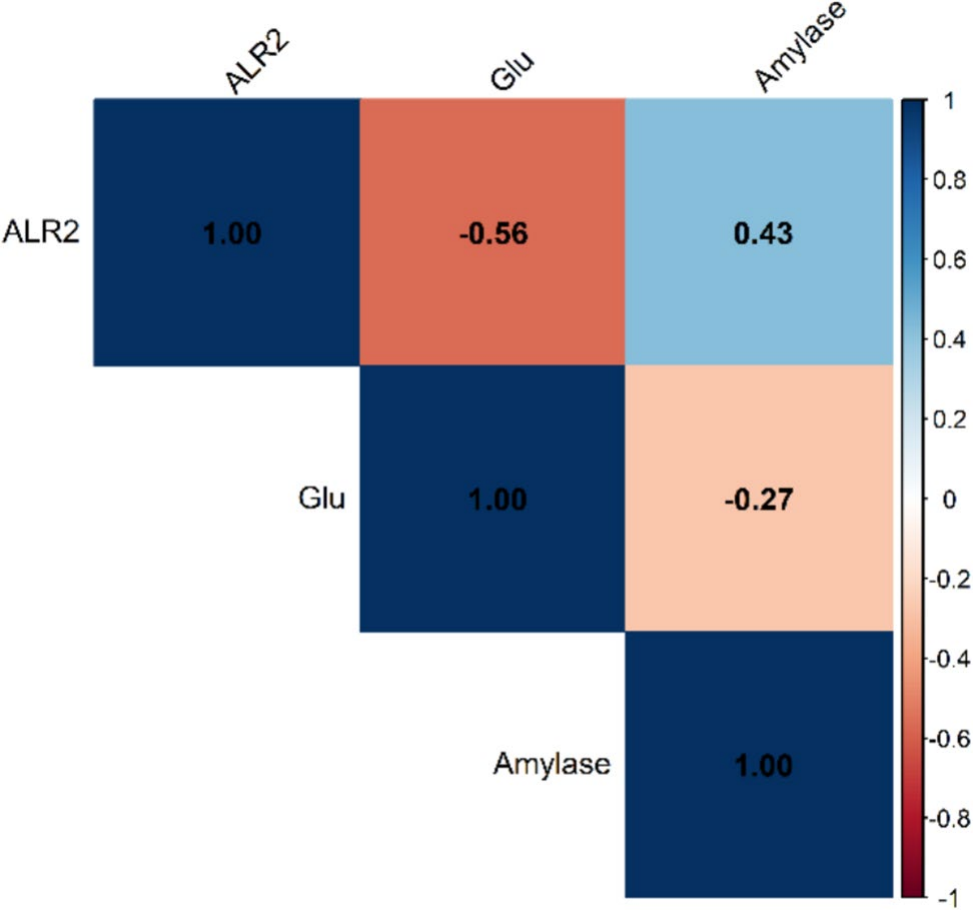
Pearson correlation heatmap illustrating the relationships between the IC_50_ values obtained for ALR2, α-glucosidase (Glu), and α-amylase inhibition across compounds **1a–1l**

To further explore the multitarget behavior of the compound series, a correlation analysis of IC_50_ values across ALR2, α-glucosidase, and α-amylase inhibition was performed (Fig. 5). The analysis revealed a moderate negative correlation between ALR2 and α-glucosidase inhibition (*r* = − 0.56), indicating that structural features favorable for ALR2 inhibition are not necessarily optimal for α-glucosidase inhibition. In contrast, a moderate positive correlation was observed between ALR2 and α-amylase inhibition (*r* = 0.43), suggesting partially shared determinants of binding affinity for these two targets. Finally, the weak negative correlation between α-glucosidase and α-amylase inhibition (*r* = − 0.27) indicates that these enzymes exhibit distinct structural requirements for ligand binding. Overall, these results support the interpretation that the present scaffold displays multitarget inhibitory activity while maintaining enzyme-specific structure–activity relationships.

### Molecular docking and molecular dynamics study

#### Molecular docking analysis

Docking-derived binding free energies were converted to K_i_ values and compared with experimental data (Table 1). Excellent agreement was obtained: R^2^ = 0.99 (α-glucosidase), R^2^ = 0.98 (ALR2), and R^2^ = 0.96 (α-amylase).

The series **1a–1l** showed close theoretical and experimental K_i_ values across all targets. Notable examples include **1h** (α-glucosidase: K_i_ = 1.48 μM vs. 1.34 μM), **1i** (ALR2: K_i_ = 0.49 μM vs. 0.49 μM), and **1j** (α-amylase: K_i_ = 1.36 μM vs. IC_50_ = 1.36 μM). Minor deviations fall within the expected uncertainty of docking and experiment.

Taken together, the strong correlations between K_i_, K_i(exp)_ and IC_50_ across all three targets confirm that the docking calculations reliably reproduce the experimental structure–activity relationships. This agreement validates the docking protocol and supports the credibility of the binding modes and interaction patterns discussed above and further refined by the MD simulations. To assess the reliability of the docking protocol, the redocking of the co-crystallized ligands from the crystal structures used as receptors was performed, wherever a well-defined active-site inhibitor was available. The ALR2 inhibitor IDD594 (ligand code LDT) from PDB ID 1US0 and pyroglutamic acid (PCA) from PDB ID 1B2Y (α-amylase) were extracted, prepared as ligands, and redocked into their native binding sites using the same AutoDock-GPU parameters and grid definitions as for the test compounds. The heavy-atom RMSD values between crystallographic and redocked poses were 1.15 Å for IDD594 and 1.69 Å for PCA, which are within the commonly accepted threshold (≤ 2.0 Å) for a successful reproduction of the experimental binding mode.

In the case of the α-glucosidase structure (PDB ID 3WY1), the bound ligand PRU ((3R,5R,7R)-octane-1,3,5,7-tetracarboxylic acid) is a crystallization additive rather than a canonical active-site inhibitor, and does not occupy the catalytic sugar-binding subsites in an analogous fashion to physiological substrates or inhibitors. Therefore, PRU was not used for quantitative redocking validation, and instead the simulations relied on benchmarking with the reference inhibitor ACR (Table 2).

**Table 2:** Binding energies (Δ*G*, kcal/mol) and binding constants K_i_ (μM) from docking, experimental K_i_ and IC_50_ (μM)

Compound	α-Glucosidase			ALR2			α-Amylase		
	Δ*G*	*K*_i_	*K*_i(exp)_	Δ*G*	*K*_i_	*K*_i(exp)_	Δ*G*	*K*_i_	IC_50_
**1a**	− 7.43	3.55	3.08	− 7.95	1.48	1.48	− 7.56	2.85	2.86
**1b**	− 7.45	3.43	3.44	− 8.06	1.23	1.22	− 7.42	3.61	3.63
**1c**	− 7.91	1.58	1.47	− 7.78	1.97	1.96	− 7.06	6.64	6.03
**1d**	− 7.69	2.29	2.28	− 6.59	14.67	10.63	− 7.61	2.62	2.62
**1e**	− 7.76	2.03	2.04	− 7.05	6.75	6.50	− 6.71	11.98	13.08
**1f**	− 7.44	3.49	3.47	− 7.34	4.14	4.22	− 7.01	7.22	4.68
**1 g**	− 7.44	3.49	3.47	− 6.78	10.65	10.55	− 7.71	2.21	2.46
**1 h**	− 7.95	**1.48**	**1.34**	− 6.70	12.19	11.94	− 7.94	1.50	1.51
**1i**	− 7.32	4.28	4.24	− 8.60	**0.49**	**0.49**	− 7.49	3.21	3.25
**1j**	− 7.09	6.31	6.26	− 7.60	2.67	2.73	− 8.00	**1.36**	**1.36**
**1 k**	− 7.75	2.07	2.05	− 7.75	2.07	2.09	− 7.41	3.67	3.67
**1 l**	− 7.43	3.55	3.26	− 7.90	1.61	1.61	− 7.16	5.60	5.73
**EPR**	/	/	/	− 7.47	3.32	3.28	/	/	/
**ACR**	− 6.64	13.5	12.6	/	/	/	− 6.87	9.15	10.0
R^2^		0.99			0.98			0.96	
	Δ*G*	RMSD		Δ*G*	RMSD		Δ*G*	RMSD	
**PCA**	− 3.78	1.69		/	/		/	/	
**IDD594**	/	/		−8.55	1.15		/	/	

*ALR2* aldose reductase *α-Glu* α-glucosidase, *Ki* predicted Ki, *Ki(exp)* experimental Ki, *R2* coefficient correlation

Comparison of binding energies shows that the most favorable derivatives bind as strongly as, or more strongly than, the co-crystallized ligands and the reference drugs. For ALR2, the redocked crystallographic inhibitor IDD594 exhibits a binding free energy of − 8.55 kcal/mol, which is essentially identical to that of the best derivative **1i** (Δ*G* = − 8.60). By contrast, the reference drug epalrestat shows a weaker docking score of − 7.47 kcal/mol, indicating that 1i is both docked and experimentally more potent than epalrestat. For α-glucosidase, compound 1 h achieves a binding free energy of − 7.95 kcal/mol, clearly more favorable than ACR (Δ*G* = − 6.64 kcal/mol) and vastly more favorable than PCA (− 3.78 kcal/mol). Likewise, for α-amylase, the most active derivative **1j** shows a docking score of − 8.00 kcal/mol, which is substantially more favorable than that of ACR (Δ*G* = − 6.87 kcal/mol). Overall, the best unnatural amino acid compounds (**1h, 1i, 1j**) exhibit binding energies and K_i_/IC_50_ values that are comparable to or better than those of the co-crystallized ligands and the reference inhibitors, in line with the excellent correlation between docking and experimental data.

#### Docking poses and interactions

**Fig. 6:**
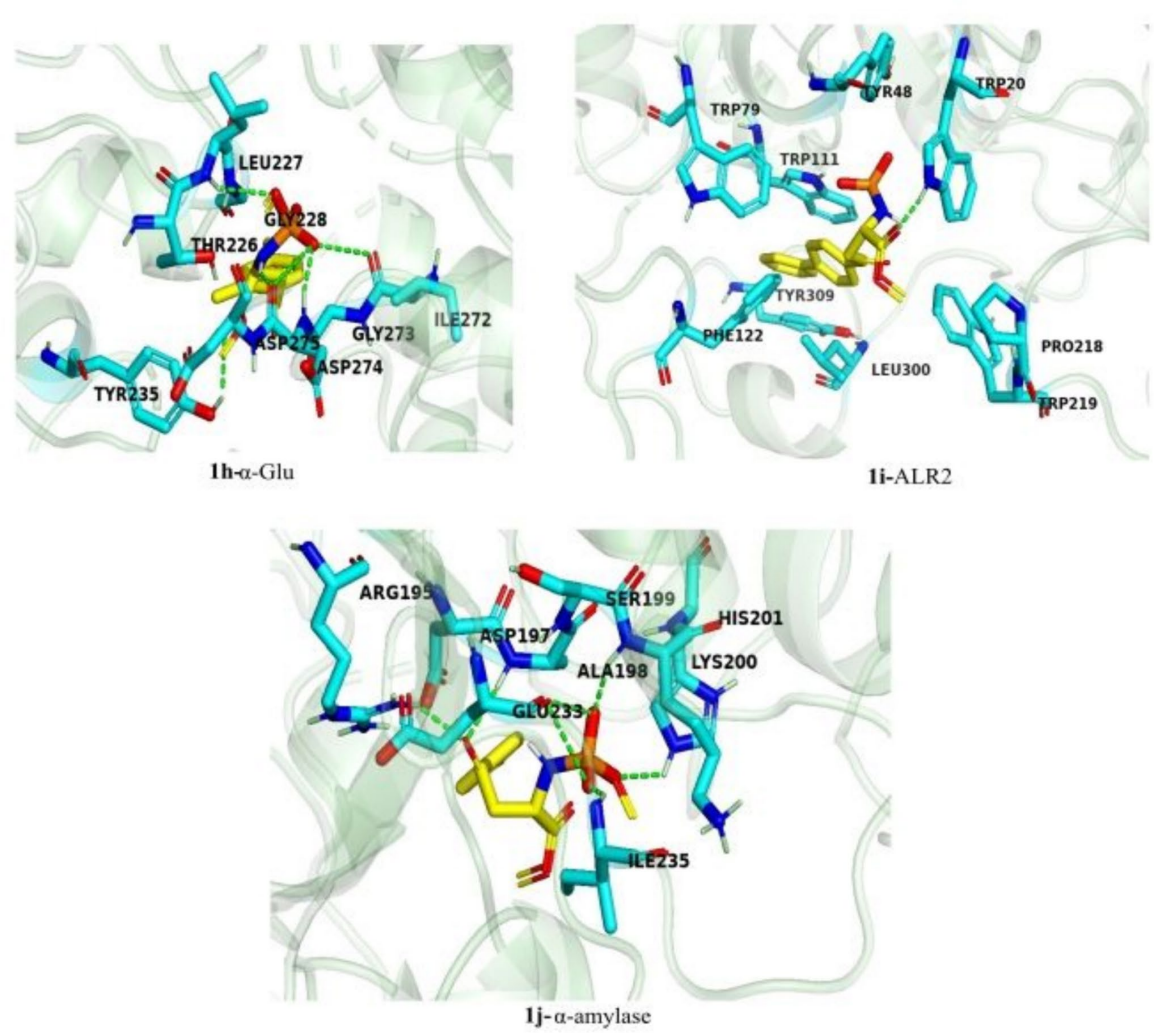
Docking poses for the most stable conformation in three receptors. (Top-left: **1h**-α-Glu; Top-right: **1i**-ALR2; Bottom: **1j**-α-Amylase. Key residues and hydrogen bonds (green dashed lines) are labeled)

Based on the calculated binding energies (Table 2), the best-ranked complexes selected for detailed inspection were **1h**–α-Glu, **1i**–ALR2, and **1j**–α-amylase. Their most stable docking poses are shown in Fig. 6. In all three cases, the ligands fit well into the catalytic pockets of the corresponding enzymes and establish several key hydrogen bonds and hydrophobic contacts.

In the **1h**–α-Glu complex, the ligand is anchored in the GH31 active site close to the catalytic acid/base residues, forming hydrogen bonds with GLY228, ASP274, ASP275, and GLY273, and additional contacts with THR226, LEU227, ILE272, and TYR235 (Fig. 6, top left). This pattern is consistent with the sugar-binding subsite and catalytic machinery described for related GH31 α-glucosidases (Shen et al. 2015). For **1i**–ALR2, the ligand occupies the classical anionic “specificity pocket” of ALR2, where it forms hydrogen bonds with TRP20 and polar groups near PHE122, while its biphenyl system engages in pronounced π–π and hydrophobic interactions with TYR48, TRP79, TRP111, TRP219, and PHE122, complemented by van der Waals contacts with LEU300 and PRO218 (Fig. 6, top right). These residues are known to be critical for ALR2 inhibitor binding in high-resolution complexes such as IDD594 (Howard et al. 2004; Türkeş et al. 2022a, b; Güleç et al. 2025). In the **1j**–α-amylase complex, the ligand spans the catalytic pocket and forms hydrogen bonds with ASP197, ARG195, GLU233, LYS200, ILE235, and SER199 (Fig. 6, bottom). The aromatic/halogenated ring is oriented toward a hydrophobic subpocket flanking the canonical ASP197–GLU233–HIS201 catalytic triad, similar to the binding mode observed for carbohydrate and non-carbohydrate inhibitors of α-amylase (Nahoum et al. 2000; Taslimi et al. 2018). These contacts indicate that **1j** can efficiently stabilize the active site and obstruct access of the natural substrate. For ALR2 and α-amylase, comparison of the docked poses of **1i** and **1j** with the redocked co-crystallized ligands IDD594 and PCA/ACR highlights (Figure S1) that our derivatives occupy the same catalytic pockets and share key interactions with the catalytic and specificity residues, while additionally exploiting hydrophobic and halogen contacts in adjacent subpockets (Howard et al. 2004; Nahoum et al. 2000; Taslimi et al. 2018).

Based on the presented results, the docking poses indicate that the selected ligands engage complementary interactions with key catalytic and structural residues in all three receptors, in agreement with their low binding energies and experimental inhibitory constants.

The differences in inhibitory potency observed among ALR2, α-glucosidase, and α-amylase can be rationalized by the structural differences in their active sites. ALR2 possesses a hydrophobic specificity pocket enriched with aromatic residues such as TRP79, TRP111, TRP219, and TYR48, which favors ligands capable of forming stabilizing π–π stacking and hydrophobic interactions. Accordingly, compounds bearing extended aromatic substituents show enhanced binding affinity toward ALR2. In contrast, α-glucosidase and α-amylase contain more polar catalytic grooves adapted for carbohydrate substrates, where ligand binding is largely governed by hydrogen bonding and polar interactions. The N-methoxysulfonyl β-ketoester scaffold combines aromatic groups capable of hydrophobic interactions with polar functional groups able to participate in hydrogen bonding. This dual interaction capability likely explains the multitarget inhibition profile of the compounds and the observed variations in enzyme selectivity among the three targets.

#### Molecular dynamics simulation

To further evaluate the stability of the docked complexes in explicit solvent, 100 ns MD simulations were performed for **1h**–α-Glu, **1i–**ALR2 and **1j–**α-Amylase. Global stability was assessed using the RMSD of the C–Cα–N backbone atoms, while local flexibility and overall compactness were monitored by RMSF and radius of gyration (Rg), respectively.

**Fig. 7:**
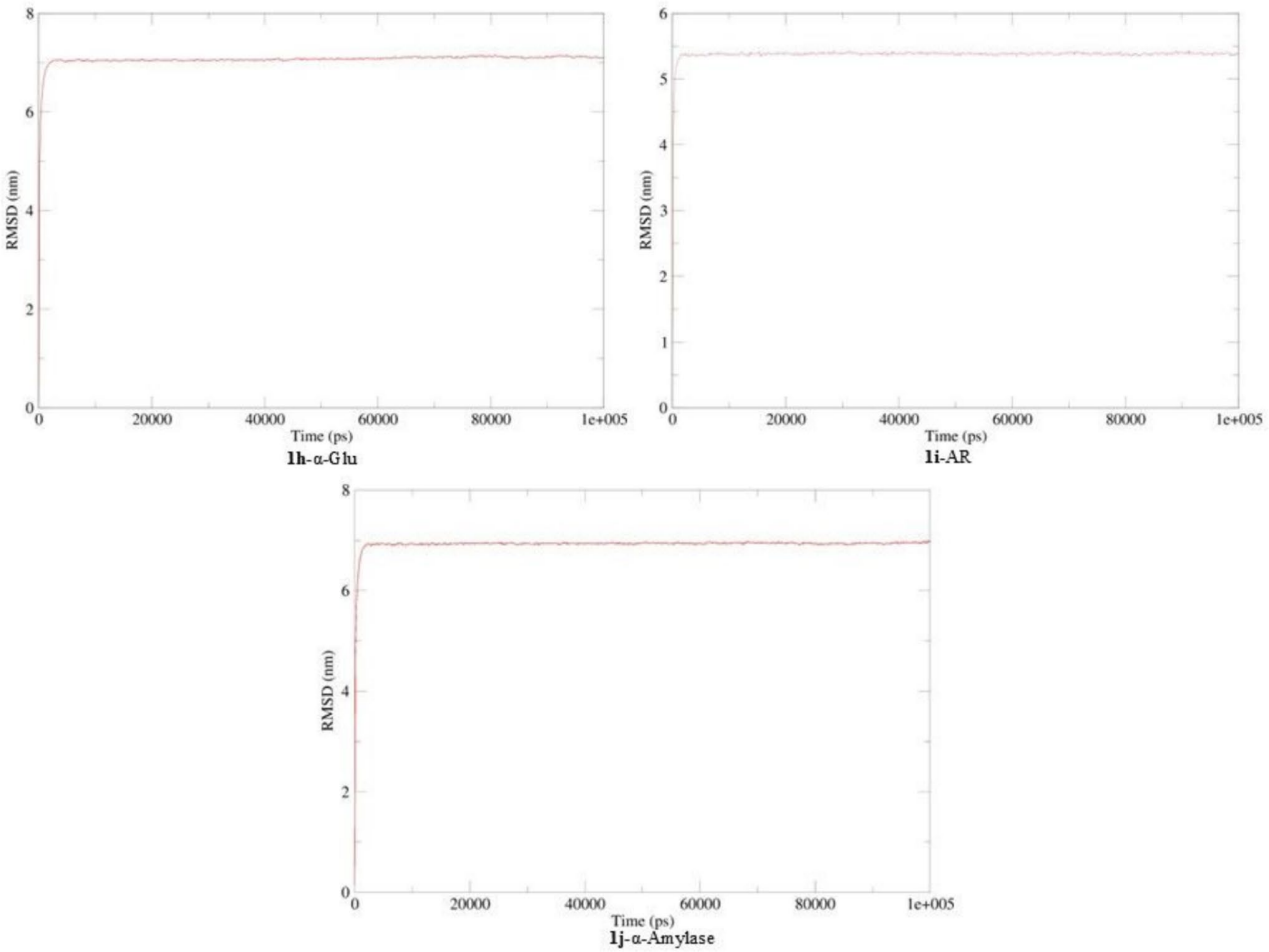
Plot of root mean square deviation (RMSD) of C–Cα–N backbone vs. simulation time for solvated investigated receptors in complex with the three candidate compounds during 100 ns molecular dynamics simulations

The RMSD values over time are presented in Fig. 7. All three complexes show a rapid increase in RMSD during the initial equilibration phase (~ 0–5 to 10 ns), followed by a plateau. After this period, the RMSD values fluctuate around nearly constant averages without large jumps, indicating that the receptor–ligand complexes remain structurally stable over the full 100 ns and that the ligands do not dissociate from the binding sites. In the present analysis the focus was on the backbone RMSD of the entire protein–ligand complexes (Fig. 7). The relatively high absolute RMSD values (~ 5–7 nm) predominantly reflect the overall size of the systems and the flexibility of peripheral loops and terminal regions, rather than large structural rearrangements in the vicinity of the active sites. Importantly, once the initial equilibration phase is over, no further systematic drift is observed and the RMSD traces remain at a well-defined plateau, supporting the view that the three protein–ligand complexes attain a stable dynamical regime on the simulated timescale.

**Fig. 8:**
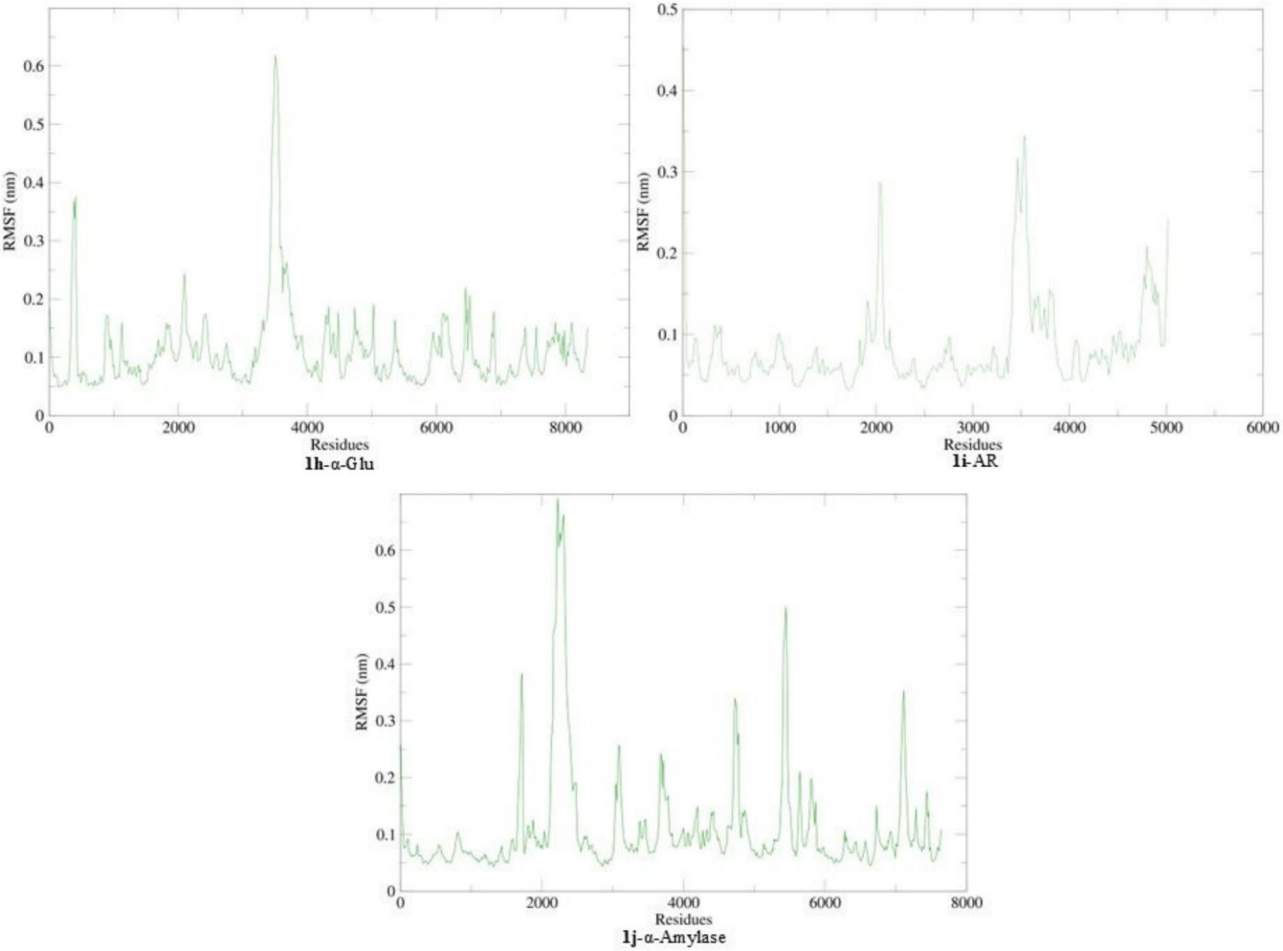
The root mean square fluctuation (RMSF) values of receptors in complex with the investigated candidate compounds were plotted against residue numbers

The RMSF (Fig. 8) profiles reveal that most residues in all three enzymes fluctuate within ~ 0.05–0.20 nm, typical of stable globular proteins. Higher RMSF peaks (> 0.4–0.6 nm) are mainly associated with flexible loops and terminal regions distant from the binding pockets. In contrast, residues directly involved in ligand binding such as ASP274/ASP275/GLY228/GLY273 in α-glucosidase, TYR48, TRP79, TRP111, TYR309 in ALR2, and ASP197, GLU233, HIS201, ARG195, LYS200 in α-amylase—display relatively low RMSF values. This suggests that the binding sites remain rigid and well stabilized by the ligands, supporting the docking results.

**Fig. 9:**
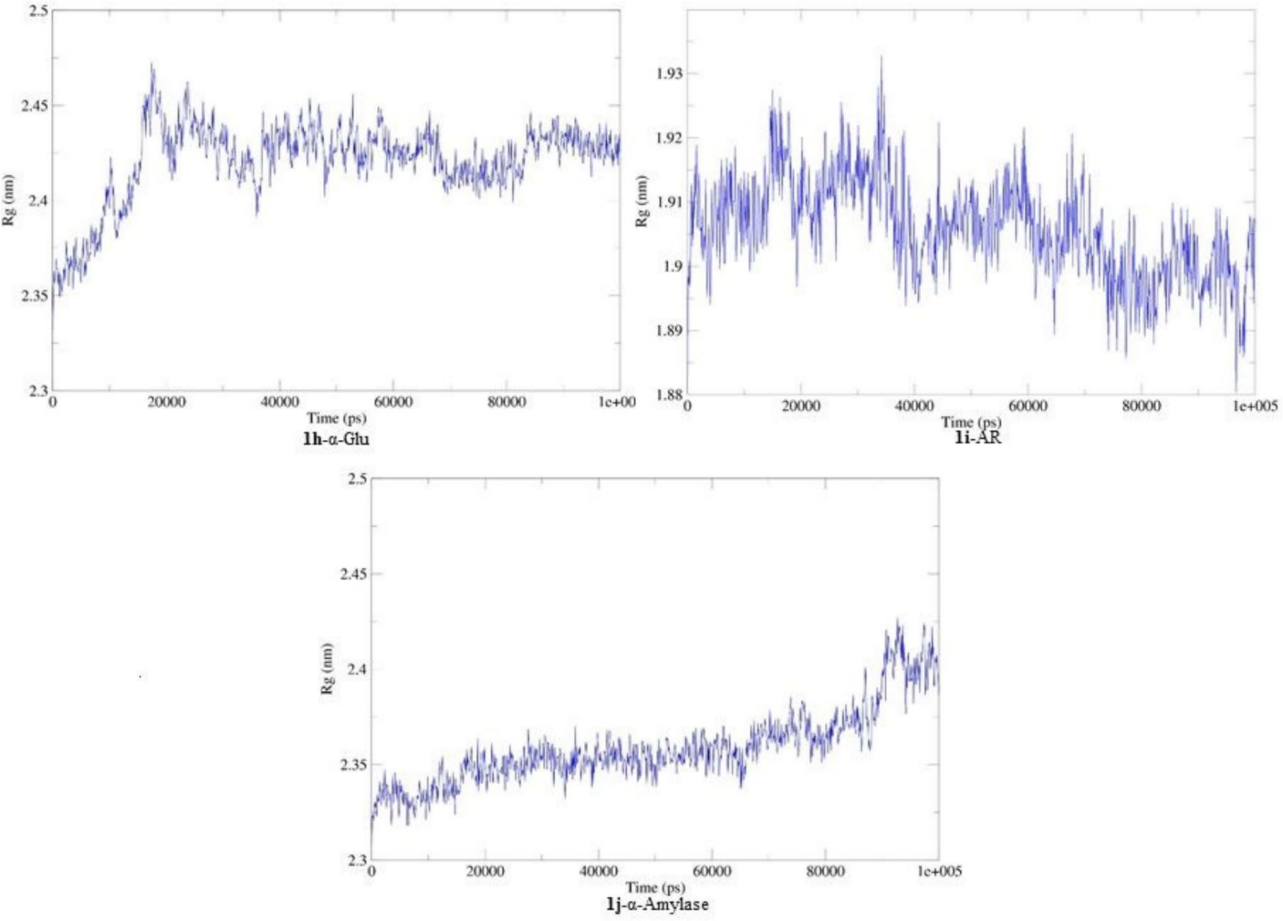
Plot of radius of gyration (Rg) during 100 ns MD simulation of the investigated receptors in complex with the three candidate compounds

Rg **(**Fig. 9) values for all three receptors remain within narrow ranges (approximately 2.35–2.45 nm for α-glucosidase and α-amylase, and ~ 1.89–1.93 nm for ALR2) throughout the simulations, with no systematic increase that would indicate unfolding. For the **1j–**α-amylase complex, a slight rise in Rg toward the end of the simulation points to minor rearrangements of surface loops rather than disruption of the protein core. Overall, the global compactness of the proteins is preserved, and ligand binding does not induce major structural perturbations.

Based on the present results, molecular docking and MD simulations consistently show that the investigated ligands form stable complexes with α-glucosidase, ALR2, and α-amylase. The excellent correlation between experimental IC_50_/K_i(exp**)**_ and docking-derived K_i_ further validates the reliability of the computational protocol and supports the proposed binding modes and interaction patterns observed in Figs. 7–9.

**Fig. 10:**
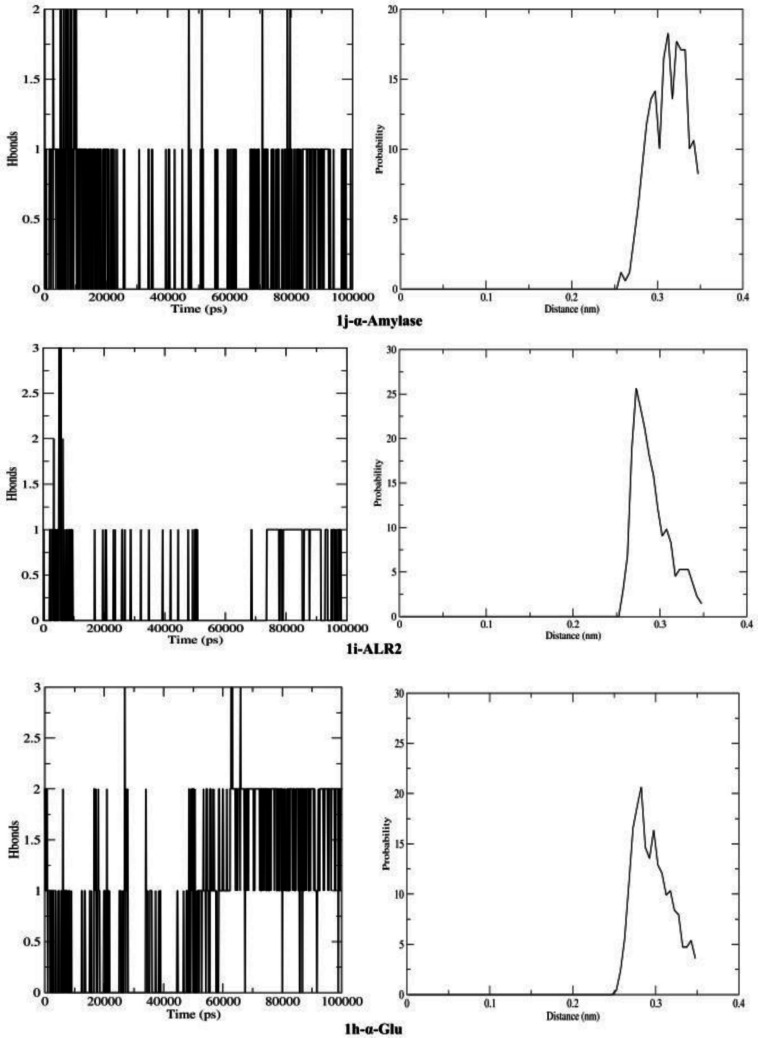
Hydrogen-bond analysis for the ligand–protein complexes

The time evolution and distance distributions of hydrogen bonds between each ligand and the corresponding protein are shown in Fig. 10. In the **1j**–α-amylase complex, the ligand maintains predominantly one hydrogen bond throughout the 100 ns simulation, with intermittent formation of a second contact. For **1i**–ALR2, the number of hydrogen bonds fluctuates mostly between zero and one, with only short-lived events in which a second hydrogen bond is formed. In contrast, the **1h**–α-glucosidase complex shows a clear strengthening of the polar interaction network after approximately 50 ns, stabilizing at one to two simultaneous hydrogen bonds and occasionally reaching three. In all three systems, the corresponding donor–acceptor distance distributions are narrowly centered around 0.27–0.32 nm, which is characteristic of well-formed hydrogen bonds. Together, these results indicate that the key polar contacts identified by docking are dynamically maintained during the 100 ns MD simulations and support the stability of the binding modes of **1h**, **1i**, and **1j**.

Despite the promising multitarget inhibitory profiles observed in the present study, certain structural features of the investigated scaffold may present limitations from a drug-development perspective. In particular, the β-ketoester moiety is known to be susceptible to enzymatic hydrolysis and metabolic transformation under physiological conditions, potentially leading to reduced in vivo stability and altered pharmacokinetic behaviour. Similarly, sulfamate-containing functionalities may undergo metabolic cleavage or phase-I/phase-II biotransformations, which could influence systemic exposure and bioavailability. These aspects represent a potential limitation of the current scaffold and should be considered in future optimization studies. Accordingly, further investigations including microsomal stability assays, metabolic profiling, and in vivo pharmacokinetic evaluation will be necessary to determine whether the observed in vitro potency can be translated into pharmacologically stable therapeutic candidates.

## Conclusion

This study presents a systematic experimental and computational evaluation of aryl-substituted unnatural *N*-methoxysulfonyl β-ketoester derivatives as multitarget inhibitors of ALR2, α-glucosidase, and α-amylase, three enzymes critically involved in post-prandial hyperglycaemia and diabetes-related complications. By integrating enzyme kinetics, SAR analysis, multivariate PCA, molecular docking, and molecular dynamics simulations, clear structure-driven trends governing potency, selectivity, and binding stability were elucidated. The enzymatic assays revealed pronounced enzyme-specific SAR patterns. ALR2 inhibition was strongly enhanced by planar aromatic expansion and conformational rigidity, with the biphenyl analog **1i** emerging as the most potent ALR2 inhibitor (K_i_ :0.493 μM), outperforming EPR. In contrast, α-glucosidase and α-amylase preferentially accommodated sterically compact yet hydrophobically enriched substituents, as exemplified by **1h** and **1j**, which showed superior activity relative to ACR. These contrasting trends highlight the distinct steric and electronic requirements of the three enzymatic targets and explain the observed selectivity profiles. PCA and contour SAR analyses further confirmed that steric factors dominate ALR2 binding, whereas α-glucosidase inhibition is primarily driven by steric accommodation within a broader hydrophobic pocket, with secondary electronic contributions. The excellent agreement between experimental and docking-derived binding constants (*R*^2^ :0.96–0.99), together with 100 ns molecular dynamics simulations demonstrating stable ligand–enzyme complexes, validates the reliability of the proposed binding modes under dynamic conditions. It should be noted that the β-ketoester functionality may be susceptible to enzymatic hydrolysis under physiological conditions. Therefore, while the present study establishes the in vitro multi-enzyme inhibitory potential of the scaffold, further metabolic stability and in vivo investigations are required to assess its translational applicability.

## Supplementary Information

Below is the link to the electronic supplementary material.

ESM 1 (DOCX 173 KB)

## Data Availability

All source data for this work (or generated in this study) are available upon reasonable request.

## Declarations

### Generative AI

Our research team utilized AI-assisted tools solely for language enhancement and readability improvement during the manuscript preparation process.
