# Supplementary material for: Unnatural amino acid compounds as potent multi-target inhibitors of aldose reductase, α-glucosidase, and α-amylase: integrated in vitro, SAR, and molecular dynamics insights
Source: Naunyn Schmiedebergs Arch Pharmacol. 2026 Mar 25;399(9):13385–404. doi: 10.1007/s00210-026-05249-1 (PMC13357411; doi:10.1007/s00210-026-05249-1)
Supplement: Supplementary file 1 — (DOCX 173 KB) [file 210_2026_5249_MOESM1_ESM.docx]

**Supplemantary file**

**Unnatural N-Methoxysulfonyl β-Ketoester Derivatives as Potent Multi-Target Inhibitors of Aldose Reductase, α-Glucosidase and α-Amylase: Integrated In Vitro, SAR and Molecular Dynamics Insights**

Serpil Gerni^a*^, Cansu Öztürk^a^, Songül Bayrak^a^, Yeliz Demir^a, b*^, Ufuk Atmaca^a^, Dejan Milenković^c^, Dušan Dimić^d^, Ömer İrfan Küfrevioğlu^a^


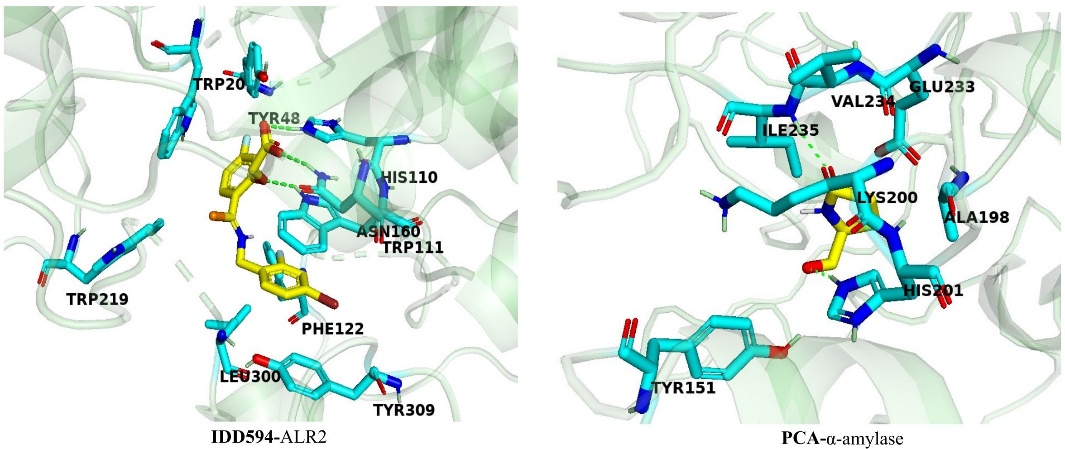


**Figure S1.** Redocked poses of the native reference ligands in the active sites of (left) human aldose reductase with IDD594 (PDB ID: 1US0) and (right) human pancreatic α‑amylase with PCA (PDB ID: 1B2Y), showing key hydrogen bonds and hydrophobic contacts with catalytic and specificity residues used to validate the docking protocol.
